# Inter-task transfer of prism adaptation depends on exposed task mastery

**DOI:** 10.1038/s41598-020-62519-5

**Published:** 2020-03-30

**Authors:** Lisa Fleury, Damien Pastor, Patrice Revol, Ludovic Delporte, Yves Rossetti

**Affiliations:** 1 INSERM U1028 CNRS UMR 5292, ImpAct Team, Lyon Neuroscience Research Center (CRNL), 69500 Bron, France; 2 “Mouvement et Handicap” platform, Neurological Hospital, Hospices Civils de Lyon, 69500 Bron, France; 3 Claude Bernard University of Lyon 1, 69100 Villeurbanne, France

**Keywords:** Cognitive neuroscience, Cognitive neuroscience, Motor control, Motor control

## Abstract

The sensorimotor system sets up plastic alterations to face new demands. Terms such as adaptation and learning are broadly used to describe a variety of processes underlying this aptitude. The mechanisms whereby transformations acquired to face a perturbation generalize to other situations or stay context-dependent remain weakly understood. Here, we compared the performance of hand pointing vs throwing to visual targets while facing an optical shift of the visual field (prismatic deviation). We found that the transfer of compensations was conditioned by the task performed during exposure to the perturbation: compensations transferred from pointing to throwing but not at all from throwing to pointing. Additionally, expertise on the task performed during exposure had a marked influence on the amount of transfer to the non-exposed task: throwing experts (dart players) remarkably transferred compensations to the pointing task. Our results reveal that different processes underlying these distinct transfer properties may be at work to face a given perturbation. Their solicitation depends on mastery for the exposed task, which is responsible for different patterns of inter-task transfer. An important implication is that transfer properties, and not only after-effects, should be included as a criterion for adaptation. At the theoretical level, we suggest that tasks may need to be mastered before they can be subjected to adaptation, which has new implications for the distinction between learning and adaptation.

## Introduction

The transfer of motor transformations remains a crucial issue both in the fields of cognitive neuroscience of action^1^ and neurorehabilitation of movement disorders^2^. Imagine a patient being able to transfer motor compensations acquired during an ideal, unique rehabilitation session onto all other daily life situations. Then, the aim for therapists would be to solicit sensorimotor plasticity processes implying transformations that can generalize beyond the context in which they were developed^3^.

Humans are remarkably capable of producing smooth and precise movements despite continuously varying demands and fluctuating internal resources. The plasticity that characterizes our nervous system encompasses the capacity to acquire new motor program and the capacity to modify existent pattern to face new conditions. Bastian relates these definitions respectively to learning and adaptation^2–4^. As such, skill learning is related to the establishment of a ‘new control policy’ while adaptation refers to the ‘recalibration of an existing control policy’^5^. Practically, learning is characterized by error reduction curves and associated with transformations that are stored and directly available in the appropriate context: individuals can switch between acquired patterns of movement depending on the specific situation. By contrast, although it also involves an error reduction curve, adaptation specifically implies the presence of compensatory after-effects (i.e. the presence of behavioral changes once the perturbation ceased)^3,4^. Few theoretical proposals have been made to clarify the terminology of learning and adaptation^2–4,6,7^, and there seems to be no consensus on this issue. In addition, only few specific empirical research is available on this distinction^5,8^. Consequently, these terms are widely used indiscriminately and interchangeably in sensorimotor plasticity literature. In an attempt to offer empirical bases on the distinction between learning and adaptation, we hypothesized that the presence of generalization beyond the exposure context may provide interesting clues about which process is predominantly used in a given situation. Patterns of transfer deliver clues about the nature of transformations that occurred in the central nervous system to face a given perturbation^1,9^. We therefore explored whether transfer properties could distinguish between processes involved in the compensations set up during exposure to a visual distorsion relatively to the expertise level on the exposed task.

Prism exposure is a classic and efficient paradigm that allows to study the implication of sensorimotor plasticity processes, respectively named as strategic recalibration and sensory realignment^4,8,10^. In a typical prism exposure protocol, subjects are actively exposed to a shift of the visual field induced by prism spectacles while performing a pointing task at least until performance regains baseline level. Then, the compensatory after-effects are measured once the prisms are explicitly removed to assess visuo-motor compensations toward untrained locations (i.e. spatial generalization)^11^. Caution for specific realignment assessment include pointing to untrained locations and explicitly removing the distorting glasses (e.g. Weiner *et al*.^12^). Recalibration allows to quickly reduce errors but not to generalize compensations. However, realignment enables to set up new body parameters and thus lead to a potential transfer of after-effects beyond the task context^13^. It is usually accepted that the respective contribution of strategic and realignment components of the compensation varies as a function of trial number, and that longer exposure increases the contribution of the sensory realignment^14,15^.

Numerous studies have investigated the generalization of prism after-effects and contrasted results are drawn. Several authors argue that transformations are specific to the context in which the participant was exposed to the perturbation. For example, after-effects would remain relatively specific to the velocity of movement trained^16^, the starting position, and effector^17^ or the task pattern^18,19^. However, other studies described alterations that transferred beyond the task context: to non-learned locations^20,21^ or to other effectors^22–26^. Much variability in the similarity between exposure and after-effect testing conditions can be found across these studies. According to the classical view of prism adaptation, proper adaptation should give rise to modifications that remain visible when the exposure context has been removed^4,6,7^. As such, cross-transfer between two tasks should provide a strong measure of context-independent adaptation by subtracting the part of after-effects potentially linked to exposure-contextual cues.

This study aimed to compare the transfer of visuo-motor transformations acquired during prism exposure between two oddly mastered tasks: throwing and pointing, the main two tasks used in the classical “prism adaptation” literature. Pointing is the most extensively used and is a casual, overlearned task associated with stable and precise performance. In contrast, throwing is far less practiced in everyday life, highly projectile dependent (e.g. dart vs basketball), usually with low accuracy constraints, and generally not fully mastered unless it is specifically trained. Despite their apparent similarities, these two aiming tasks are associated with very different level of mastery and therefore variability in performance^27,28^. Our prediction was that the asymmetrical degree of mastery for these tasks should yield to an asymmetrical pattern of transfer. First, we showed that these two tasks produce highly comparable mean error-reduction curve and after-effects although variability was much higher for throwing than pointing. Crucially the transfer test revealed a unidirectional transfer from pointing to throwing and null transfer from throwing to pointing (Experiment 1). Second, throwing experts (dart players) transferred prism compensations from throwing to pointing. These findings suggest that expertise determines preference for context-independent processes to face sustained perturbation (Experiment 2). Finally, we attempted to investigate the mechanisms underlying the unidirectional transfer and showed that kinematics of pointing movements uncover potential mechanistic explanations for our results (Experiment 3).

## Results

### Experiment 1: Influence of the task performed during prism exposure on transfer

**Figure 1:**
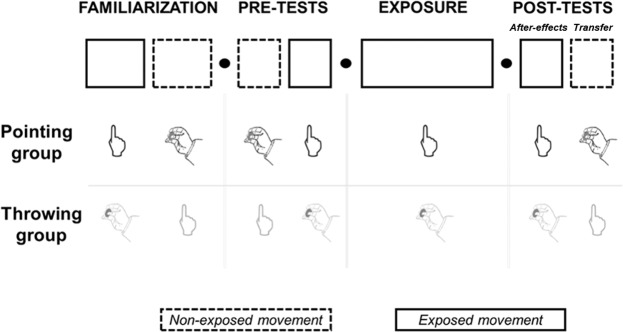
Different steps of the experimental protocol. Solid-line boxes represent the steps in which participants performed the exposed task and dotted-line boxes are related to the non-exposed task. For each step of the experiment, tasks performed are illustrated for the pointing and the throwing groups. Early tests (specific to experiment 3) are not represented.

In experiment 1, we randomly assigned 24 participants to two groups according to the goal-directed task realized during exposure to the optical shift. The pointing group performed finger pointing during exposure while the throwing group threw small spheres during exposure. We first familiarized participants on both tasks. Then we measured participants’ performances on both tasks (pre-tests). Participants were then asked to wear prismatic glasses that induced a 10 degrees shift of the visual field toward the right while performing the exposed task (throwing or pointing). Immediately after exposure, we removed prisms and assessed after-effects with the exposed task (classical measure of adaptation) and then with the non-exposed task (transfer). The different steps of the experiment are illustrated in Fig. 1 and the specific conditions of each step are summarized in Table 1. Performance was measured as the lateral deviation between the index endpoint (pointing) or ball impact (throwing) and the aimed target and are reported in degrees (see Methods section for a precise description of measurements). Trial-by-trial average endpoint errors during the whole experiment are presented in Supplementary Figure 1 (Supplemental information). Statistical analysis results are detailed in Tables 2–5. Means are reported together with standard deviations.

**Table 1:** Summary of the specific conditions for each step of the experiment.

	Familiarization	Pre-tests	Exposure	Early tests	Post-tests
Prisms	OFF	OFF	**ON**	OFF	OFF
Visual Feedback	Available	**None**	Available	**None**	**None**
Targets	Central	Central or Right	Central	Central or Right	Central or Right
Number of trials	2*30	2*20	60	2*20	2*20

Note: early tests are specific to experiment 2.

**Table 2:** Statistical analysis: rMANOVAs during familiarization, and pre-tests in Experiments 1, 2, 3.

ANOVAs Familiarization and Pre-Tests										
*Experiment 1*										
		**Throwing group**		**Pointing Group**		***Significant Effect***	***dl***	***F***	***p***	*Post-Hoc significant*
		**Throwing task**	**Pointing task**	**Pointing task**	**Throwing task**					
		***(A)***	***(B)***	***(C)***	***(D)***					
Familiarization	*Mean*	0,19 ± 1,06	0,08 ± 0,22	0,12 ± 0,23	−0,01 ± 0,93	—	*No significant effect*			
	*Variance*	4,84 ± 3,25	0,25 ± 0,15	0,27 ± 0,43	3,82 ± 2,40	**Task**	(1, 22)	49.37	**10**^**−7**^	A, D > B, C**
Pre-Tests	*Mean*	−0,01 ± 2,40	−0,47 ± 2,40	−0,23 ± 1,68	−0,77 ± 1,37	—	*No significant effect*			
***Experiment 2***										
		**Throwing Comfortable Experts**		**Throwing Uncomfortable Experts**		***Significant Effect***	***dl***	***F***	***p***	***Post-Hoc significant***
		**Throwing task**	**Pointing task**	**Throwing task**	**Pointing task**					
		***(E)***	***(F)***	***(G)***	***(H)***					
Familiarization	*Mean*	0,16 ± 0,74	0,09 ± 1,16	0,21 ± 0,80	0,05 ± 0,14	—	*No significant effect*			
	*Variance*	1,48 ± 0,57	0,08 ± 0,04	2,00 ± 0,00	0,08 ± 0,05	Task	(3,26)	20.93	**10**^**−4**^	A, D, G > B, C, E, F, H**
Pre-Tests	*Mean*	−0,43 ± 1,54	0,32 ± 1,46	3,77 ± 1,83	−0,50 ± 0,07	Task*Group	(3,26)	3.37	0.03	G > E, F, H
***Experiment 3***										
		**Throwing group**		**Pointing Group**		***Significant*** ***Effect***	***dl***	***F***	***p***	***Post-Hoc significant***
		**Throwing task**	**Pointing task**	**Pointing task**	**Throwing task**					
		***(A)***	***(B)***	***(C)***	***(D)***					
Familiarization	*Mean*	0,18 ± 0,72	0,83 ± 0,57	0,99 ± 0,44	0,26 ± 0,65	Task	(1, 18)	15.59	**10**^**−4**^	B, C > A, D*
	*Variance*	5,41 ± 3,68	0,28 ± 0,22	0,32 ± 0,53	9,19 ± 6,55	Task	(1, 18)	34.21	**10**^**−5**^	A, D > B, C**
Pre-Tests	*Mean*	0,68 ± 1,25	0,06 ± 1,01	0,62 ± 0,88	0,84 ± 1,3	—	*No significant effect*			

Notes: HSD Tukey’s were used for experiment 1 and 2. LSD Fisher’s were used for experiment 3. Both were significant at p < 0.05(*) and p < 0.01 (**). Only significant effects are reported. Mean values for each group are reported on the left side of the table in degrees (mean) and degrees² (variance) together with standard deviations.

**Table 3:** Statistical analysis: T-tests against zero during pre-tests, early-tests and post-tests in Experiments 1, 2, 3.

T-Tests against zero: Pre-Tests, Early-Tests, Post-Tests					
**Experiment 1**					
***Group***	***Task***	***Mean (degrees) ± SD***	***t***	***dl***	***p***
***Pre-tests***					
Throwing	Exposed task (Throwing)	−0,01 ± 2,40	−0.01	11	0.99
	Non exposed task (Pointing)	−0,47 ± 2,21	−0.72		0.48
Pointing	Exposed task (Pointing)	−0,23 ± 1,68	−0.47		0.64
	Non exposed task (Throwing)	−0,77 ± 1,37	−1.95		0.08
***Post-tests***					
Throwing	After-effects (Throwing)	−4,87 ± 1,81	−9.33	11	**10**^**−6**^
	Transfer (Pointing)	0,05 ± 0,97	0.19		0.85
	Transfer Ratio (%)	−6,02 ± 28,90	−0.72		0.48
Pointing	After-effects (Pointing)	−4,43 ± 1,09	−13.99		**10**^**−6**^
	Transfer (Throwing)	−1,80 ± 1,00	−5.49		**10**^**−5**^
	Transfer Ratio (%)	44,39 ± 30,33	5.07		**10**^**−4**^
**Experiment 2**					
***Group***	***Task***	***Mean (degrees) ± SD***	***t***	***dl***	***p***
***Pre-tests***					
Comfortable Experts	Exposed task (Throwing)	−0,43 ± 1,54	−0.54	3	0.62
	Non exposed task (Pointing)	0,32 ± 1,46	0.45		0.69
Uncomfortable Experts	Exposed task (Throwing)	3,77 ± 1,83	2.9	1	0.21
	Non exposed task (Pointing)	−0,50 ± 0,07	−10		0.06
***Post-tests***					
Comfortable Experts	After-effects (Throwing)	−4,47 ± 0,86	−10.41	3	**10**^**−3**^
	Transfer (Pointing)	−1,72 ± 0,62	−5.49		**0.01**
	Transfer Ratio (%)	31,61 ± 12,99	4.86		**0.01**
Uncomfortable Experts	After-effects (Throwing)	−6 ± 0,34	−24.59	1	**0.02**
	Transfer (Pointing)	−0,12 ± 0,9	−0.19		0.87
	Transfer Ratio (%)	1,65 ± 14,92	0.16		0.91
**Experiment 3**					
***Group***	***Task***	***Mean (degrees) ± SD***	***t***	***dl***	***p***
***Pre-tests***					
Throwing	Exposed task (Throwing)	0,68 ± 1,25	1.71	9	0.12
	Non exposed task (Pointing)	0,06 ± 1,01	0.32		0.85
Pointing	Exposed task (Pointing)	0,62 ± 0,88	2.21		0.06
	Non exposed task (Throwing)	0,84 ± 1,3	0.41		0.07
***Early Tests***					
Throwing	Exposed task (Throwing)	−2,82 ± 1,71	−5.23	9	**10**^**−4**^
Pointing	Exposed task (Pointing)	−3,79 ± 2,02	−5.91		**10**^**−4**^
***Post-tests***					
Throwing	After-effects (Throwing)	−4,88 ± 1,63	−9.48	9	**10**^**−5**^
	Transfer (Pointing)	−0,16 ± 0,87	−0.6		0.56
	Transfer Ratio	6,69 ± 38,24	0.55		0.59
Pointing	After-effects (Pointing)	−5,07 ± 1,08	−9.05		**10**^**−5**^
	Transfer (Throwing)	−3,08 ± 2,19	−4.45		**10**^**−3**^
	Transfer Ratio (%)	54,71 ± 53,78	3.22		**0,01**

**Table 4:** Statistical analysis: T-tests between groups during post-tests, in Experiments 1, 2, 3.

Statistical analysis of Post-tests - Experiments 1, 2, 3						
***Experiment 1***						
	**Pointing group**	**Throwing group**	***t***	***dl***	***p***	
After-effects	−4,43 ± 1,09	−4,87 ± 1,81	0.72	22	0.48	
Transfer	−1,80 ± 1,00	0,05 ± 0,97	4.62		**10**^**−4**^	
Experiment 2*						
	**Comfortable Experts**	**Uncomfortable Experts**	***R²***	***dl***	***F***	***p***
After-effects	−4,47 ± 0,86	−6 ± 0,34	0.08	3.26	0.78	0.51
Transfer	−1,72 ± 0,62	−0,12 ± 0,9	0.51	3.26	9.13	**10**^**−4**^
***Experiment 3***						
	**Pointing group**	**Throwing group**	***t***	***dl***	***p***	
Early-Tests	−5,07 ± 1,08	−4,88 ± 1,63	0.24	18	0.81	
After-effects	−5,07 ± 1,08	−4,88 ± 1,63	0.24		**10**^**−3**^	
Transfer	−3,08 ± 2,19	−0,16 ± 0,87	3.9		**10**^**−3**^	

^*^For experiment 2, reported values refer to a 1-way ANOVA comparing experts groups and throwing and pointing control groups. Post-Hoc analyses revealed significant differences between comfortable Experts and throwing controls group (p < 0.05).

**Table 5:** Statistical analysis: rMANOVAs during exposure in Experiments 1, 2, 3.

rMANOVAs Exposure										
**Experiment 1**										
	Block 1	Block 2	Block 3	Block 4	Block 5	Block 6	dl	F	p	Significant effects
Throwing group	5,02 2,40	2,91 ± 1,98	2,13 ± 1,42	1,65 ± 1,20	1,11 ± 0,58	1,01 ± 0,85	1, 22	36.46	10^−7^	Block
Pointing Group	4,29 ± 2,91	2,38 ± 2,95	1,57 ± 1,85	1,13 ± 1,04	0,78 ± 0,55	0,68 ± 0,37				
**Experiment 2**										
True Experts	5,38 ± 1,06	3,64 ± 1,70	1,88 ± 1,08	1,20 ± 0,83	0,96 ± 0,92	0,88 ± 0,94	3, 26	46.02	10^−7^	Block
False Experts	4,28 ± 1,33	2,72 ± 0,51	2,03 ± 0,19	0,89 ± 0,01	0,96 ± 0,38	0,47 ± 0,10				
**Experiment 3**										
Throwing group	4,16 ± 2,10	3,54 ± 1,40	1,24 ± 1,15	1,18 ± 0,88	0,90 ± 0,84	0,95 ± 0,80	1, 18	16.27^1^	10^−7^	Group^1^, Group*Block^2^
Pointing Group	2,10 ± 0,81	1,77 ± 1,12	1,30 ± 0,74	1,21 ± 0,58	1,31 ± 0,54	1,31 ± 0,60		6.21^2^		

#### No difference during familiarization except in variability

**Figure 2:**
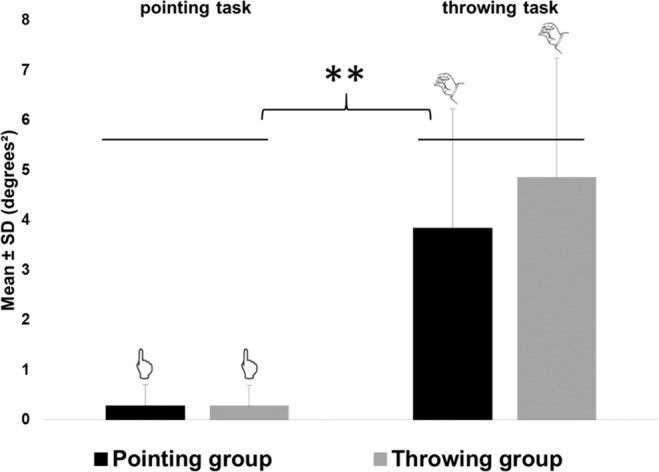
Experiment 1: mean variances during familiarization. Mean group variances are represented in black for the pointing group and in grey for the throwing group, respectively on the left for the pointing task and on the right for the throwing task. Error bars refers to standard deviations. **means p < 0.01.

Average individuals’ performance, i.e. mean endpoint errors, was similar between groups (F(1,22)=0.13; p = 0.71) and close to zero (non-significant t-test against zero for both groups and both tasks). However, the mean variability of endpoints errors was much larger for throwing movements (mean variance = 4.84 ± 3.25 degrees² for the throwing group; 3.83 ± 2.40 degrees² for the pointing group) than for pointing movements (mean variance = 0.26 ± 0.16 degrees² for the throwing group; 0.27 ± 0.43 degrees² for the pointing group) in both groups (significant task effect, F(1,22) = 49.37; p < 10^−7^), see Fig. 2.

#### No difference in baseline performance

Participants showed the same average performance (close to zero) regardless of the task performed (F(1,22) = 0.16; p = 0.69). The mean variability of throwing movements was also about 8 times larger than pointing movements for both groups.

#### Similar error reduction during prism exposure

Both groups exhibited similar error reduction curves. A repeated-measure ANOVA (rmANOVA) comparing mean individuals’ endpoint errors between groups (Pointing and Throwing) and across blocks (1 to 6) revealed a significant effect of Block (F(1,22) = 36.46; p < 10^−7^) but no main effect of Group and no interaction. Thus, during exposure subjects reduced their errors similarly regardless of the task performed during exposure.

Noticeably, error reduction was not complete at the end of the exposure for both groups. Indeed, the mean endpoint error during late exposure (10 last trials) was significantly greater than during late familiarization (10 last trials) in both group (1.01 ± 0.85 vs 0.27 ± 1.05 in the throwing group, t(11) = −2.68, p < 0.05; 0.68 ± 0.38 vs 0.12 ± 0.23 in the pointing group, t(11)  = −4.42, p < 0.05).

#### Similar after-effects

**Figure 3:**
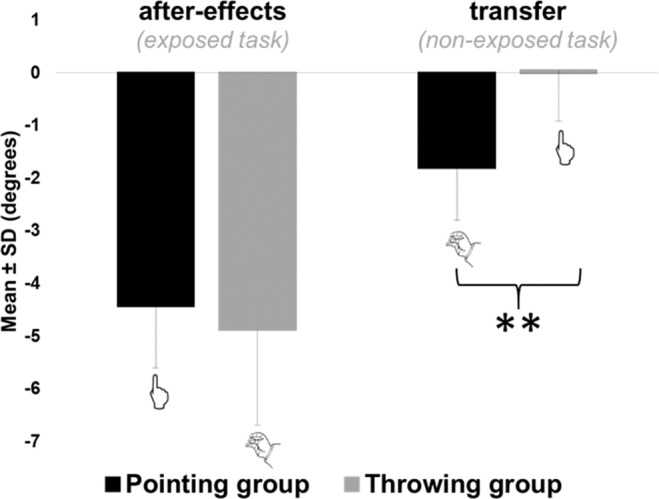
Experiment 1: mean endpoints errors during post-tests. Mean group endpoint errors during post-tests are represented in black for the pointing group and in grey for the throwing group, respectively on the left for after-effects (i.e. endpoint errors on the exposed task) and on the right for transfer (i.e. endpoint errors on the non-exposed task). Error bars refers to standard deviations. **means p < 0.01.

After-effects as well as transfer values are reported after subtracting mean baseline performances (pre-tests) for each subject. Participants showed significant errors opposite to the prismatic deviation in both Throwing (mean = −4.51 ± 1.68 degrees; t_zero_(11) = −9.26, p < 10^−6^) and Pointing group (mean = −4.43 ± 0.09 degrees; t_zero_ (11) = −13.99, p < 10^−7^). Participants showed the same amount of after-effects on the exposed movement, regardless the task performed during exposure (t(22) = 0.72, p = 0.48), see Fig. 3.

#### Unidirectional transfer

The pointing group showed a significant transfer of compensations to the throwing task (mean = −1.76 ± 1.00 degrees, t_zero_(11) = −6.08, p < 10^−5^) while the Throwing group showed no transfer to the pointing task (mean = 0.01 ± 0.92 degrees, t_zero_(11) = 0.04, p = 0.97), see Fig. 3. Thus, the amount of transfer was greater in the pointing group compared to the throwing group (t(22) = 4.62, p < 10^−4^), see Fig. 3. We quantified the transfer of compensation as the percent of the average endpoint errors on the non-exposed movement divided by the average endpoint error on the exposed movement. The Pointing group demonstrated as much as 44.39 ± 29.65% transfer whereas the Throwing group did not exhibit any transfer (−6.02 ± 25.67%). Results from experiment 1 highlight a strictly unidirectional transfer of compensations from pointing to throwing but not from throwing to pointing. However, we did not find any apparent dissimilarities on baseline performances, neither on error reduction during exposure nor on the amount of after-effects on the exposed task. One possible explanation is that adaptation would not transfer from the far space of throwing to the near space of pointing. An alternative explanation relates to motor variability. Both groups showed a higher variability on the throwing task compared to the pointing task at all stages of the experiment, which confirms that throwing was less mastered than pointing in our sample. To specifically test for this mastery hypothesis, we recruited 6 high-level French dart players as experts in throwing (Experiment 2). If mastery rather than target distance explains the occurrence of transfer, then throwing experts should exhibit some transfer of after-effects from the throwing task to the pointing task.

### Experiment 2 - Influence of the expertise degree on the transfer

Six high-level dart-players with practice experience ranging from eight to forty years (Expert group) completed the experimental protocol following the same steps as the Throwing group in experiment 1. They were exposed to the prismatic deviation while performing throwing and tested for the transfer to the pointing task. Statistical analysis results are detailed in Tables 2–5.

#### Similarities between laboratory throwing task and dart throwing movement

Two expert participants reported very poor scores of task comfort and movement similarity relative to their usual dart throwing movements (see Table 6). Therefore, we divided our Experts group into two sub-groups: comfortable Experts (n = 4) and uncomfortable Experts (n = 2). Results from the two expert groups were compared to the results obtained in experiment 1. Trial-by-trial mean endpoint errors are represented in Supplementary Figure 2.

**Table 6:** Throwing experts characteristics.

Expert	Years of practice	Best rank	Comfort	Similarity
1	40	Reg (3rd)	2	2
2	8	Nat (2nd)	−3	−2
3	15	Nat (9th)	−1	−1
4	15	Nat (12th)	−1	2
5	15	Reg (10th)	−3	−3
6	13	Reg (20th)	3	1

Note: comfort and similarity scores refer to the self-rate performed by dart players so as to assess the likeness between dart throwing and our experimental throwing task. Comfort and Similarity were rated from from −3 (“not at all comfortable” and “very different”) to 3 (“very comfortable” and “very similar”).

#### Motor variability associated with expertise

**Figure 4:**
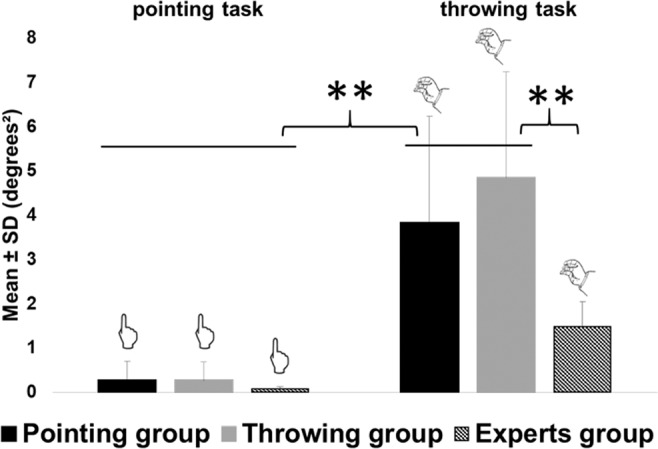
Experiment 2: mean variances during familiarization. Mean group endpoint errors during post-tests are represented in black for the pointing group, in grey for the throwing group, in hatched for the throwing expert group, respectively on the left for the pointing task and on the right for the throwing task. Experts group refer to the comfortable experts only. Uncomfortable experts’ results are not represented. Error bars refers to standard deviations. **means p < 0.01.

During familiarization trials, comfortable Experts showed a significantly lower variability on the throwing task compared to the control groups (Throwing and Pointing groups in experiment 1) (mean = 1.48 ± 0.57 degrees²; F(3,26) = 20.96, p < 10^−4^). This stronger consistency in throwing movements compared to the throwing control group is related to the expertise on these throwing movements despite the dissimilarities between dart throwing and experimental throwing (see Fig. 4). Uncomfortable Experts showed an intermediate level of variability (mean = 2.00 ± 0.00 degrees²) that was also lower compared to the throwing control group.

#### No difference in baseline

Average endpoint errors were around zero and were not significantly different between Experts (both Comfortable and Uncomfortable) and controls (Pointing and Throwing groups) (F(3,26) = 1.16, p = 0.34).

#### Similar error reduction and after-affects

Expertise was not associated with a faster error reduction during exposure. Indeed, experts showed no difference in their performance while they were exposed to the prismatic deviation compared to the pointing and throwing control groups. The rMANOVA (Group*Block) revealed only a significant effect of blocks (F(3,26) = 46.02, p < 10^−7^).

**Figure 5:**
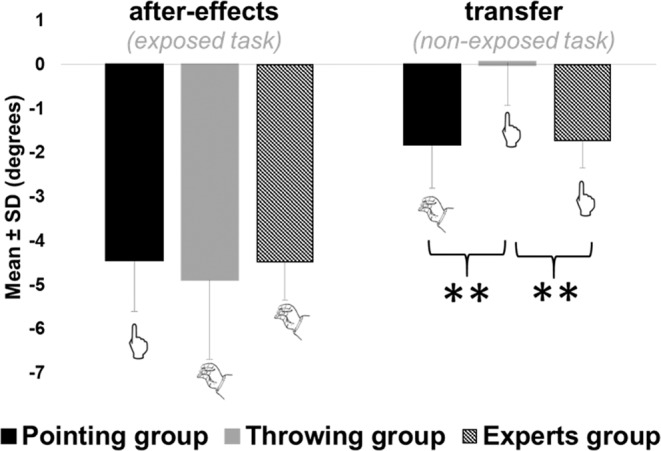
Experiment 2: mean endpoints errors during post-tests. Mean group endpoint errors during post-tests are represented in black for the pointing group, in grey for the throwing group, in hatched for the throwing experts group and respectively on the left for after-effects (i.e. endpoint errors on the exposed task) and on the right for transfer (i.e. endpoint errors on the non-exposed task). Experts group refer to the comfortable experts only. Uncomfortable experts’ results are not represented. Error bars refers to standard deviations. **means p < 0.01.

Concerning after-effects, comfortable Experts exhibited the same amount of after-effects on the trained task than the two other controls group (Pointing and Throwing) (mean = −4.48 ± 0.86 degrees; F(3,26) = 0.78, p < 0.51), see Fig. 5. Uncomfortable experts also showed a comparable amount of after-effects (mean = −6.00 ± 0.34 degrees).

#### Reciprocal transfer in experts

Except for variability, Experts did not display differences compared to the Pointing and Throwing control groups for the previous classical variables of prism adaptation. However, our results crucially revealed a significant presence of after-effects on the non-exposed task (i.e. pointing) in the comfortable Experts group (t(3) = −5.49, p = 0.01), see Fig. 5. In fact, comfortable Experts showed transfer to the task they did not practice during exposure (i.e. pointing) that was comparable to the Pointing group, and obviously greater than Throwing control group (mean = −1.72 ± 0.62 degrees, (F(3,26) = 9.13, p < 10^−4^). However, uncomfortable Experts did not show any significant transfer of after-effects to the non-exposed task (mean = −0.12 ± 0.90 degrees).

#### Relation between motor variability and transfer

**Figure 6:**
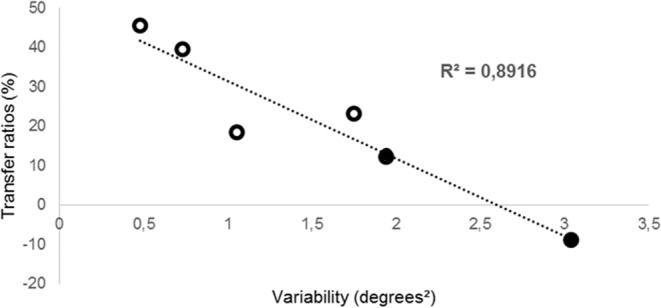
Experiment 2: correlation between transfer ratios and variability of throwing movements in the experts group. Variability refers to the mean variances of the ten last familiarization throwing trials for each subject. Both comfortable (empty marks) and uncomfortable (solid marks) experts are represented.

As explained previously, during our laboratory throwing task, two expert participants were not able to fully reproduce the feelings and comfort associated to their usual dart-practice. Consistently, they displayed a greater variability than other experts in the group, meaning that their dart expertise was not entirely beneficial during our laboratory throwing task. Moreover, these two participants showed no transfer to the pointing task. In addition, a highly significant correlation (R² = 0.89) was found between variability during the last ten familiarization throwing movements and the transfer ratio in the whole expert group, indicating that the more variable the participants, the less they were able to transfer compensations to the non-exposed task, see Fig. 6. However, this correlation remained far from significance in the novice control throwing group.

As a matter of fact, the second experiment revealed that mastery of the exposed task (throwing) promotes the transfer of acquired compensations to the non-exposed task (pointing). Thus, context-independent processes seem to be involved to compensate for a given perturbation while performing a mastered motor task. Transfer exhibited by comfortable experts also allowed us to rule out the idea that transfer may operate only from near to far space. However, the previous experiments do not allow to understand the physiological mechanisms underlying the link between expertise and transfer. A third experiment was conducted in order to better understand the strict unidirectionality of transfer between throwing and pointing. In this experiment, we analyzed the kinematics of pointing movements performed during pointing task in order to investigate whether different phases of the trajectory may be differentially affected by prism adaptation (see O’Shea *et al*.^8^).

### Experiment 3: Mechanisms underlying the unidirectional transfer between throwing and pointing

Our specific aim was to investigate whether differential after-effects would provide a predictive variable for transfer. We also provide kinematic analyses of pointing trajectories in order to better understand the mechanisms underlying the absence of transfer in the throwing group. The design of the experiment was the same except that we introduced early post-tests and measured pointing kinematics with motion capture (see Online methods for details). Statistical analysis results are detailed in Tables 2–5, 7, 8.

**Table 7:** Kinematic alterations of pointing movements: T-tests between Throwing and Pointing groups in Experiments 3.

Kinematic alterations: T-Tests (Experiment 3)					
**Movement directions during Pre-tests (both groups)**					
	**Pointing group**	**Throwing group**	**t**	**dl**	**p**
Initial direction	−6,37 ± 7,02	−8,42 ± 3,95	0.8	18	0.43
Intermediate direction	2,62 ± 3,14	2,38 ± 2,08	0.19		0.85
Terminal direction	5,35 ± 5,42	3,62 ± 2,68	0.86		0.4
**Movement directions alterations during Post-tests (both groups)**					
	**Pointing group**	**Throwing group**	**t**	**dl**	**p**
Initial direction	−4,84 ± 3,25	−3,13 ± 1,48	−1,51	18	0.15
Intermediate direction	−6,25 ± 3,58	−6,13 ± 4,09	−0,07		0.94
Terminal direction	−5,69 ± 5,76	2,37 ± 2,72	−4,00		**10**^**−4**^
**Movements direction alterations during Early-Tests and Post-Tests (Pointing group)**					
	**Early Tests**	**Post-tests**	**t**	**dl**	**p**
Initial direction	−5,55	−4,84	−0,45	9	0.66
Intermediate direction	0,12 ± 3,58	−6,25 ± 3,58	3.89		**10**^**−3**^
Terminal direction	−3,77 ± 7,91	−5,69 ± 5,75	1.92		0.49

**Table 8:** Temporal dynamics of after-effects: T-tests between Throwing and Pointing groups in Experiments 3.

Temporal dynamics of after-effects in early tests and post-tests: T-Tests (Experiment 3)					
**Early-tests**					
	**Pointing group**	**Throwing group**	**t**	**dl**	**p**
5 first trials	−4,49 ± 2,01	−3,36 ± 2,57	1.1	18	0.29
5 last trials	−3,45 ± 1,99	−1,17 ± 1,85	2.66		**0.01**
**Post-tests**					
	**Pointing group**	**Throwing group**	**t**	**dl**	**p**
5 first trials	−5,69 ± 1,72	−5,92 ± 1,89	0.29	18	0.77
5 last trials	−5,05 ± 1,99	−2,56 ± 2,87	2.24		**0.03**

**Figure 7:**
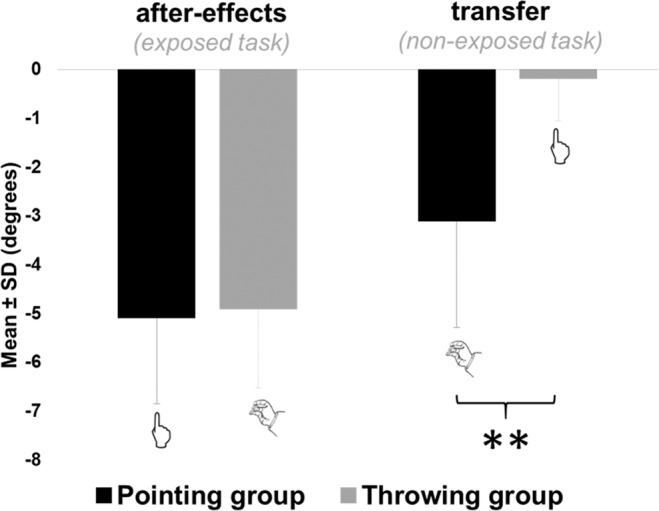
Experiment 3: mean endpoints errors during post-tests. Mean group endpoint errors during post-tests are represented in black for the pointing group, in grey for the throwing group, and respectively on the left for after-effects (i.e. endpoint errors on the exposed task) and on the right for transfer (i.e. endpoint errors on the non-exposed task. Error bars refers to standard deviations. **means p < 0.01.

As expected, the overall pattern of results was similar to experiment 1, i.e. similar average results through familiarization, pre-tests, exposure and post-tests. The transfer test also confirmed a unidirectional transfer from pointing to throwing but not from throwing to pointing (see Fig. 7).

A significant effect of Group on error reduction during exposure was observed during the first two blocks of exposure in which average endpoint errors were larger in the throwing group compared to the pointing group (4.16 ± 2.10 and 3.54 ± 1.40 degrees for block 1 and 2 in the throwing group vs 2.10 ± 0.81 and 1.77 ± 1.12 for the pointing group), which is compatible with lowering speed stress imposed on subjects (see supplementary information, Supplementary Figure 4).

As in experiment 1, a significant variability difference was found between throwing and pointing in both group during familiarization (F(1,18) = 34.25, p < 10^−5^) (see supplementary information, Supplementary Figure 3).

Concerning the early measures of compensation, no significant difference between groups were observed, which means that subjects showed apparently similar magnitude of after-effects on the exposed task after the first block of exposure independently from the trained task (t(18) = 1.62, p = 0.12).

#### Kinematics show a symmetrical transfer during initial direction

**Figure 8:**
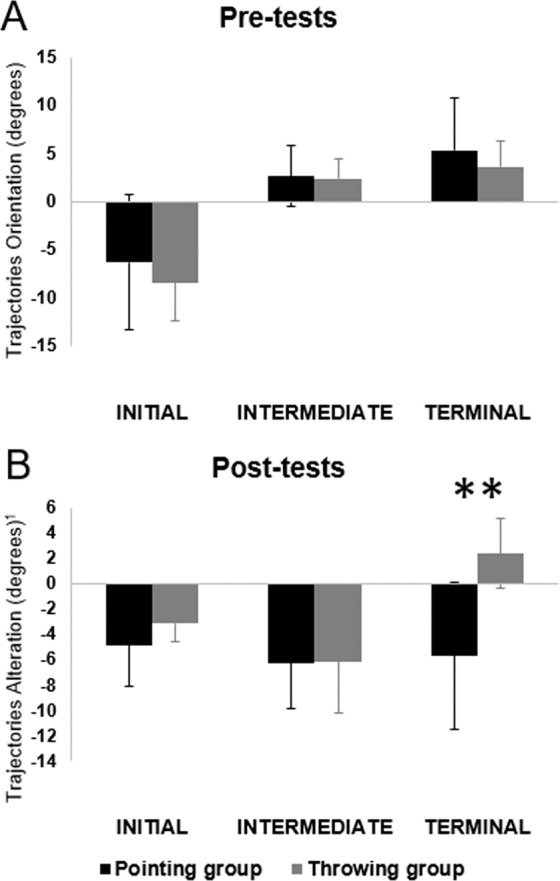
Experiment 3: mean pointing trajectory orientation and alteration during initial, intermediate and terminal) phases of movements. Comparison between pointing (in black) and throwing (in grey) groups during Pre-tests (PRE), and Post-tests (POST). **means p < *0.01. Note: for Post-tests, mean baseline trajectories orientation have been subtracted for each subject. Trajectories alterations are represented*.

Mean pointing trajectories are reported in supplementary information (Supplementary Figure 5) for Throwing and Pointing groups. Movements’ instantaneous directions were compared at the main kinematic landmarks: acceleration peak (initial direction), velocity peak (intermediate direction), and deceleration peak (terminal direction) between groups, during pre-tests, early tests and post-tests (Fig. 8). Directions were measured as the angle between instantaneous velocity vector at each peak and the straightforward line between the starting position and the target. Reported values only concern the central target. We first compared mean movement directions at each kinematic landmark during pre-tests for both groups. The results revealed that the mean movement direction at initial, intermediate and terminal phases of pointing movements did not differ between groups during Pre-Tests (t(18) = 0.8; 0.19; 0.86 respectively and p = 0.43, 0.85; 0.4. Mean values are reported in Table 7), thus the two groups initially produced comparable trajectories.

Then, we compared alteration of movements’ directions during Post-tests as the difference in movement directions between Pre-tests and Post-tests respectively for initial, intermediate, and terminal directions. After exposure, the same substantial alteration was observed during Post-tests on the pointing task in both groups for initial and intermediate directions (t(18) = −1.51; −0.07 and p = 0.15; 0.94 respectively). It suggests that a similar compensation took place in the two groups in term of initial motor commands. However, mean terminal direction of movements was altered differently in Throwing group compared to the Pointing group during Post-tests (mean = 2.37 ± 2.72 degrees for Throwing group and −5.69 ± 5.76 degrees for Pointing group, t(18) = −4.00, p < 10^−3^). This again confirms that despite apparent similarities the nature of the compensation differed between the two groups. Results showed that participants in the Throwing group were able to transfer compensations in the initial and intermediate phase of movements but corrected trajectories in the final phase of movements, which lead to an absence of transfer when endpoints are considered. This analysis may provide explanations for the crucial differences observed between transfer of the two groups that are masked on the endpoint accuracy measures.

## Discussion

In the present study we explored the cross-transfer between two manual visuo-motor tasks: throwing and pointing. Our observations yield four main findings: (1) prism exposure during pointing and throwing movements produced strikingly similar error reduction curves and after-effects after prism removal, (2) in spite of this apparent similarity, transfer of compensations was radically unidirectional, i.e. reliably present from pointing to throwing and fully absent from throwing to pointing, (3) kinematic analyses of pointing trajectories suggested plausible physiological mechanisms underlying findings 1 and 2, (4) expertise on the throwing task (dart throwers) was associated with the transfer of compensations from throwing to pointing task. Altogether we argue that a proper definition of adaptation should include transfer properties in addition to the classical after-effect criterion.

### Unidirectional transfer provides a signature for different processes

Our results show that two manual aiming tasks yield to apparently similar results in terms of pre-tests performance, error reduction during prism exposure and after-effects, which may have suggested that similar compensation took place in the two groups. According to the classical definition of adaptation, i.e. simply based on the presence of compensatory after-effects upon explicit removal of the perturbation, throwing and pointing would be considered as producing identical adaptation. In spite of this apparent evidence, a strictly unidirectional transfer occurred, which suggests that compensation resulted from different process according to the task exposed. As a matter of fact, participants performing throwing during prism exposure showed no transfer to the pointing task upon prisms removal. It suggests that during exposure, participants set up visuomotor corrections to address the visual-motor discrepancy encountered and finally managed to compensate for the deviation to return to their baseline performances. Although participants showed after-effects on the trained task (i.e. throwing), these after-effects did not transfer to the non-exposed task (i.e. pointing). This implies that the underlying processes of this compensation remained context-specific. Whereas the compensations set up during classical prism exposure can be explained in terms of changes in the alignment of spatial maps (visual head-eye and proprioceptive head-hand)^29,30^, the lack of transfer from throwing to pointing with the same limb instead suggests that compensation at work relied on motor commands rather than sensory realignment^8,10^. One fundamental implication of this result is that the classical definition of adaptation, i.e. based on the presence of compensatory after-effects, may not be sufficient to clearly outline the boundary between different processes at work, which might be related to learning and adaptation. True adaptation may actually require further specifications in terms of spatial generalization^31^ and transfer to other contexts. This finding is reminiscent of the observation that compensations acquired during exposure to force-fields do not transfer to unconstrained arm movements^32^. We may speculate that participants in our throwing group solicited a more strategic level of compensation than true low-level realignment. Since post-test conditions for the exposed movement (classical after-effects) were close from the exposure condition even upon prism removal (same task, trained target), participants after-effects may result from associative generalization (i.e. toward close conditions). Conversely, the pointing group exhibited after-effects both on the exposed task and the non-exposed task, i.e. an associative and dimensional (i.e. above initial conditions) generalization at the same time. Thereby, the pointing group seemed to show a higher implication of the realignment processes while the throwing group seemed to rely mainly on strategic processes of error correction^8^.

Our third experiment was conducted to refine our analysis and to investigate potential explanations for this unidirectional transfer. We showed that early compensations were not different between Throwing and Pointing groups and thus could not explain the differences observed in transfer capacities across groups. However, analysis of pointing movement kinematics showed that a transfer from throwing to pointing seems to be present at the initial and intermediate phases of movements but disappears during the terminal phase of movements, which leads to an absence of transfer concerning the endpoint error. In a previous study, O’shea *et al*.^8^ showed that kinematics allowed to distinguish two different error corrective processes during prism exposure: a strategic feedforward motor control process (initial part of the trajectory) and a slower feedback-driven correction process (terminal part of the trajectory). Echoing this previous study, the present analysis showed that kinematics reveal crucial information about the nature of processes involved in addition to endpoint errors. A possible explanation may be relative to the proprioceptive component in prism adaptation. The felt position of the hand is not shifted by the prism while the visually perceived location of the target is. As such, proprioceptive feedback will tend to guide the hand toward the virtual target location, away from the real target location^8,10^. It is admitted that prism exposure of pointing movements alters the felt limb position sense^13,30,33^. Our results suggest that novice participants in the throwing groups may not exhibit such change, and the available literature does not allow to know whether prism exposure by throwing produces a similar proprioceptive after-effect when throwing movements are exposed to the optical shift. Instead, they might have relied more on the feedforward motor control process, i.e. the adjustment of motor plan for the subsequent trial^8,10^. Thus, when subjects from the throwing control group made pointing movement during post-tests, they were able to correct their trajectory once proprioceptive feedback was at work, as the proprioceptive modality was not altered during prism exposure.

### Expertise enables transfer of compensations

Two differences between tasks might explain this unidirectional transfer: the space of action (near space for pointing vs far space for throwing) and the variability associated with each task. Motor variability related to throwing movement may have influenced the processes set up to compensate for the prismatic perturbation^27^. Throwing experts enable us to address these two hypotheses.

In the first experiment, both groups showed a higher variance in throwing than pointing. This can be explained by the fact that pointing is a usual and highly mastered task while throwing needs a lot of practice to be controlled. Relationships between motor variability and learning capacities have been recently studied. Some authors suggested that variability, instead of being an unwanted noisy consequence, would rather be linked to the capacity of exploring multiple motor solutions. This action exploration would be associated with a better performance in sensorimotor learning in a non-mastered manipulandum task movements^34,35^ and related to an increased interlimb transfer^36^. In our second experiment, we recruited throwing experts to investigate the influence of expertise on the transfer of visuo-motor compensations acquired during prism exposure. Experts interestingly showed a significant transfer of compensations on the non-exposed task (i.e. pointing) while the control throwing group did not. In contrast to the aforementioned studies, our results showed that the less subjects were variable during the familiarization phase on the exposed task, the better they transferred visuomotor compensations to the non-exposed task. Darts experts showed a decreased variability compared to control groups which confirms that consistency (i.e. low variability) is a suitable marker of expertise^28,37^. Moreover, we found that transfer rates are correlated with variability and expertise. Thus, expertise seems to influence the transfer properties following prism exposure. Indeed, the results suggest that a higher implication of sensorimotor adaptive processes was present in the expert group, similar to the pointing control group. The correlation between variability and transfer in the expert group may be interpreted cautiously due to the small number of experts. Nevertheless, the absence of correlation in the novice groups may suggest that an expertise threshold conditions transfer. As such, we speculate that novices have no option but opt for the establishment of a new control policy, i.e. prompt learning, in order to face a new sensorimotor situation. Conversely, sensorimotor expertise appears to be necessary to adjust an existing control policy to face a perturbation, i.e. to activate adaptation. Our results are congruent with previous findings that emphasized the influence of expertise on the processes used to face varying conditions^38,39^.

Therefore, expertise on the task performed during prism exposure seems to play a role in conditioning the transfer of after-effects to an unexposed task. An interesting question is whether expertise on the transfer task also influences the amount of transfer on this task. Healthy individuals can be considered as natural experts on the pointing task. However, their pointing expertise did not allow those who performed the throwing task during prism exposure to transfer the acquired compensations to the pointing task. Hence, we can argue that performing an over trained task during prism exposure favors “true” adaptation and thus, the transfer of visuo-motor compensations to a non-trained task. Conversely, before the exposed task is fully controlled; one relies on cognitive strategies - strategic errors reduction^40^ in a greater way and is not allowed to set up a “true” adaptation process in response to prismatic deviation.

A speculative explanation of the link between expertise and transfer relates to the role of proprioception in movement control. Kinematics analysis from experiment 3 suggest that proprioceptive modality was not altered when novice participants performed throwing under prism exposure. As such, prism exposure may have altered experts felt limb position in such a way that they exhibited transfer of after-effects over the whole pointing trajectory. Several studies showing that expertise in a given motor task modifies proprioceptive abilities already support this hypothesis (e.g. Lin, Lien, Wang, & Tsauo, 2006)^41^ and it remains to be specifically tested. Future studies measuring kinematics data for experts will allow us to test whether transfer also appears in the terminal phase of movements in expert participants.

### Implications for learning and adaptation

From a theoretical perspective, our results provide empirical arguments for the distinction of processes leading to context-dependent vs generalizable after-effects, congruent with several distinctions proposed earlier^2–6,12^. We speculate that the context-dependent process which leads to local transformations obtained during exposure to throwing may be classified as learning, while transferable compensations in a different context would pertain to adaptation. Our study supports the fact that context-dependency of elicited transformations may be crucially related to the very nature of the plastic process involved in the compensation of the visual shift produce by prisms. The fact that throwing experts, who have over-learned the throwing movements, exhibit transfer from throwing to pointing (with a magnitude comparable to the transfer exhibited by subjects from pointing to throwing) strongly supports the idea that true adaptation can occur once a visuo-motor task has been sufficiently practiced to give rise to learning. In principle, it would not seem optimal to adapt sensorimotor transformation parameters before they have been defined in a sufficiently accurate way, i.e. adaptation may not be useful until the range of adaptation falls below the variability of the task performance. As a consequence, discrepancies experienced when practicing an unmastered task during prism exposure would not be relevant enough to give rise to adaptation^42^. Therefore, one may speculate that adaptation of a given task may only take place once this task has been sufficiently acquired through learning, and further investigations will be required to further test this hypothesis.

## Conclusion

Our study compared two tasks during prism exposure, with a high level of similarity in terms of both compensations developed during the perturbation and classical after-effects. In spite of similar after-effects, transfer of compensations after the perturbation occurred only from the most mastered task to the least mastered task. This strictly unidirectional transfer was demonstrated by means of both traditional manual measurement (experiment 1) and sophisticated motion tracking technique (experiment 3). In addition, motor expertise in darts players for the exposed task appears to promote transfer capacities. In light of these results, we speculate that context-dependent learning (i.e. cognitive, strategic) processes are mainly at work to first stabilize performance in novice participants while experts use context-independent adaptation (i.e. sensorimotor) processes to face the perturbation. Thus, future full definitions of adaptation should include transfer properties in addition to after-effects.

Our study uncovered a unidirectional transfer of prism-acquired after-effects from pointing to throwing and we put forward several hypotheses (i.e. the role of proprioceptive feedbacks and the likely relationship between variability/expertise and transfer) that need to be tested. Open questions relate to the threshold of expertise needed to enable transfer, and to the neural substrates underlying such capacities. Further investigations are needed to explore how the brain switches from learning to adaptation over the course of expertise acquisition, to define optimal adaptation procedures and to design rehabilitation strategies based on the elicitation of context-independent adaptation that would transfer benefits to daily life situations.

## Methods

Our study was divided into three distinct experiments illustrated in supplementary information (Supplementary Figure 6). Every participant gave informed consent to participate in these experiments. All procedures were designed following relevant guidelines and regulations and were approved by the ethics evaluation committee of Inserm (IRB of the French Institute of medical research and Health, IRB00003888, IORG003254, FWA00005831).

### Experiment 1

#### Participants

24 healthy volunteer subjects participated in the study. They had normal or corrected to normal vision, no neurological disorder and had never experienced prisms before the experiment. Participants were asked to perform two goal-directed visuo-motor tasks –throwing and pointing – which are detailed in the further section.

Participants were divided into two groups depending on the task performed during prism exposure (which was called « exposed movement » in contrast to « non-exposed movement »). Thus, for the first group, the exposed movement was pointing while the non-exposed movement was throwing (**Pointing** group, n = 12, 6 males and 6 females, mean age = 22.58 ± 1.73 years old). For the second group, the exposed movement was throwing, and the non-exposed movement was pointing (**Throwing** group, n = 12, 6 males and 6 females, mean age = 22.16 ± 2.79 years old), see Fig. 1.

#### Experimental paradigm

Participants followed the four stages illustrated in Fig. 1 with varying tasks performed. Tasks were performed either with vision allowed (closed-loop) or without (open-loop), and toward one central or two targets (central (0°) and right (10°)). Specific conditions for each step of the experimental are detailed in this section and summarized in Table 1.

### Familiarization

The aim was to familiarize participants with both tasks and with experimental settings. They were asked to perform trials of the exposed movement then trials of the non-exposed movements. As pointing is an over-trained task, participants performed only 10 trials versus 30 trials for the throwing task. Trials were performed in a closed-loop condition (vision allowed) toward the central target.

### Pre-tests

Participants performed two blocks of ten trials of the non-exposed movement and then two blocks of ten trials of the exposed movement. Trials were performed in open-loop condition (no vision) and toward both targets in a randomized order (which was the same for all participants).

### Exposure

Participants were asked to perform six blocks of ten trials while wearing prismatic lenses that shifted the visual field ten degrees toward the right (OptiquePeter.com, Lyon). Trials were performed in a closed-loop condition (vision allowed) toward the central target at a maximal speed. Before positioning the goggles, participants were asked to keep their eyes closed. They were also instructed not to look at their own body or to move in any way (except to perform the task) while they were wearing the goggles.

### Post-tests

Once the prisms were removed, participants were asked to perform two blocks of ten trials of the exposed movement to assess after-effects. Then, they performed two blocks of ten trials on the non-exposed task to measure the transfer of after-effects. The four blocks were realized in the same conditions than during pre-tests: no vision allowed, both right and central target in a randomized order.

#### Pointing and throwing task set-ups

At the beginning of the experiment, participants were asked to sit in an adjustable and movable chair and to stay on this chair for the whole duration of the experiment. Investigators were in charge to move the participant from the throwing experimental set-up to the pointing one when it was necessary. During each transitional phase, participants were wearing eye patches to be deprived of vision and to prevent influences of environmental vision on visuo-motor compensations. The experimental set-ups for each task are detailed in the following section.

### Pointing task

Participants were sitting in front of a pointing desk, with their head on a chinrest. The starting position of the finger was situated below the chinrest, lined up with the body midline. Chinrest was used to avoid participants to see their hand starting position in order to prevent any static recalibration of the prism induced shift and thus slow down the error reduction, see supplementary information (Supplementary Figure 7).

Two targets were positioned on the pointing desk in front of participants, at a distance of 57.5 cm from their eyes. The central target (exposed target) was situated straight ahead of the participant’s body midline (0 degrees) and the right target (non-exposed target) at 10 degrees to the right. During open-loop pointing (pre, early, post-tests), no vision was allowed during the entire movement. To control visual feedback, the investigator manipulated a cover board in front of the participants. Before each trial, the investigator lowered the cover board, so the participants were able to see the targets while their index lied on the starting position. Before the beginning of the trial, the investigator lifted the cover board to prevent vision of the targets and movements. This condition was used in order to reliably measure after-effects without the subject detecting and correcting for after-effect induced biases.

During closed-loop pointing (familiarization and exposure), the investigator did not manipulate the cover board and full vision was allowed. In both conditions, participants were asked to point as quickly as possible. To control movement duration, participants were previously trained to reach the target in less than 250 ms. The starting signal was given by the investigator for each trial. A color code indicated which target to reach in open-loop conditions and a vocal « go! » was provided during closed-loop conditions (using only one target).

### Throwing task

Participants sat on a comfortable, adjustable chair in front of a vertical board, at a distance of 2 meters. They were wearing a ball-dispenser helmet illustrated in Supplementary Figure 7. Two targets were materialized on the board. The central target (exposed target) was situated forward the participant’s body midline (0 degrees) and the right target (non-exposed target) at 10 degrees to the right. All the setting was surrounded by light spots connected to a switch placed on the helmet. During open-loop throwing, participants had to pick a ball and to press down the light-switch mounted on the helmet, so as to be able to see the target. Once they initiated movement and released the switch, lights were immediately turned off so they were not able to see anymore. Thus, participants had no visual feedback concerning their movements and consequences. During closed-loop throwing (familiarization and exposure), lights were turned on and participants were able to see their movement all time. As for pointing, in both conditions, participants were asked to throw as fast and accurate as possible. The starting signal was given by the investigator for each trial: the target color to reach in open-loop conditions and « go » during closed-loop conditions.

#### Data collection

For the pointing task, graduations were present on the pointing desk, opposite to the subject. The investigator reported manually the endpoint error of each reaching trial. For the throwing task, an optoelectronic motion capture system (9 cameras, Vicon Motion Systems Ltd, Oxford, 1984) was used to record the ball impact on the vertical board for each throwing trial. Reflective markers were placed on the throwing board to identify the targets. Moreover, the projectiles were reflective themselves.

#### Data processing

Markers trajectories were recorded for each trial and filtered with a Butterworth low-band pass filter at a cut-off frequency of 6 Hz. For the throwing trials, the time-point corresponding to the contact between the projectile and the board was automatically detected. This time point was used to compute the lateral errors between the impact of the projectile and the aimed target using MatLab customized routines.

Thus, we computed performances on each trial, i.e. angular deviation between the ball impact and the aimed target.

#### Statistical analysis

Dependent variable was the endpoint lateral error for each trial, expressed in degrees. The distance between the aimed target and either the final position of the index (pointing trials) or the ball impact (throwing trials) in centimeters was converted into degrees through classical trigonometric rules, taking in account the distance between the eyes and the target. rMANOVAs (Group*Task) were performed to assess differences between groups during familiarization and pre-tests in terms of endpoints errors and variability (comparisons of variances during familiarization). A rMANOVA (Group*Block) was performed to assess differences during exposure. For post-tests measurements, mean baseline performances (during pre-tests) were subtracted to after-effects and transfer values individually for each subject so as to correct for any baseline deviation. T-tests against zero were used to identify significant alterations during post-tests (after-effects and transfer). T-tests were also to compare both group during after-effects and transfer assessment. Statistical analysis was performed on Statistica 7.1 (StatSoft Inc., Tulsa, 1984). All mean values are reported together with standard deviations.

### Experiment 2

This second experiment aimed to test the influence of expertise on the task performed during exposure on the transfer of compensations to the non-exposed task. We recruited six dart players to constitute an Experts group (6 males, mean age = 32 ± 11.22 years old). They were all practicing darts for at least 8 years and ranked at a national (or excellent regional) level. Although dart throwing presents several high similarities with our throwing experimental set up, there were some differences concerning the distance (2.37meters from the targets in dart throwing vs 2 m in our experimental protocol), the posture (standing up vs sitting facing forward), the starting position (hand seen vs hand hidden), and of course the projectile (dart vs ball). To check whether dart expertise correlated with expertise in our throwing task, we asked experts to self-rate the task comfort and the movement similarity between dart throwing and our throwing set up from −3 (“not at all comfortable” and “very different”) to 3 (“very comfortable” and “very similar”).

Then, they followed the same protocol as the Throwing group of experiment 1: they were exposed to the prismatic deviation while performing throwing and tested for the transfer of compensations on the pointing task.

Data from Experts were compared to control pointing and throwing groups whose data were recorded in experiment 1. All procedures concerning data collection and data processing were similar to procedures used in experiment 1. Statistical analysis was performed similarly to experiment 1, except that we used a 1-way ANOVA in order to test for differences between the three groups in terms of after-effects and transfer.

### Experiment 3

The aim of this experiment was to identify the possible mechanisms underlying the unidirectional transfer pointed out in the first experiment. As such, we aimed to test the early compensations set up at the beginning of exposure. The two compensatory processes involved in prism exposure are known to be associated with different timings: recalibration would occur firstly while realignment would take a longer time to be set up (see O’shea *et al*., 2017). Given these differences, we aimed to test whether the presence and the amount of early after-effects would highlight any difference in the implication of both processes. We also aimed to investigate pointing kinematics to refine our analysis of differences between groups. Our purpose was to examine pointing trajectories to shed light on potential clues appearing before the end of the movement, i.e. at initial and intermediate trajectory directions. The same general procedures and methods were used. Thereafter are detailed the changes operated.

#### Participants

We recruited 20 other healthy participants (n = 10, 5 males and 5 females, mean age = 38.3 ± 18.73 years old for the Pointing group, and n = 10, 5 males and 5 females, mean age = 37 ± 17.26 years old for the Throwing group) following the same inclusion criteria as in experiment 1.

#### Familiarization

We decided to keep to the same amount the number of trials for throwing and pointing task even though pointing is over-trained.

#### Early compensations

We can hypothesize that the amount of after-effects consecutively to the first trials during exposure may be different between the two groups, according to the task performed. In order to test for this hypothesis, we designed an experiment in which we introduced additional probe trials following the first block of exposure. Right after the ten first trials of exposure, we removed the goggles and asked participants to keep their eyes closed. Thein, they immediately performed 20 trials of the exposed movement to assess the presence of early after-effects, in an open-loop conditions (with no vision allowed during movement) and toward the central or the right target.

#### Pointing task

In experiments 1 and 2, subjects were instructed to reach the target as quickly as possible and trained to perform movement under 250 ms. In order to investigate pointing trajectories in more ecological conditions, the investigator instructed the subject to point “as fast and accurate” as possible without training them to reach under a specific movement duration. In addition, we automatized the control of visual feedbacks during open-loop condition. Participants wore electronic liquid-crystal glasses connected to a switch placed on the starting position. The participants were able to see the targets while their index finger lied on the starting position. Upon movement initiation the glasses turned opaque and subjects were deprived of visual feedback. When they went back to the starting position, the glasses turned transparent again.

#### Data collection and data processing

The motion capture system were also used to record pointing trajectories. Reflective markers were positioned on the index, the wrist and the elbow of the subject. The endpoint of each pointing movement was computed automatically (using in-house custom software written in Matlab). Movements were detected using the following thresholds: onset was defined as the point at which hand velocity exceeded 80 mm/s while offset was defined as the time-point at which velocity dropped below this threshold. After automatic detection, all trials were cross-checked visually and adjusted manually if necessary. Index endpoints were then used to obtain lateral endpoint errors from the aimed target. Only pointing movement kinematics were analyzed.

#### Statistical analysis

The statistical analysis was performed similarly to previous experiments We added T-tests to test for differences in pointing movement directions between both groups. We compared initial, intermediate, and terminal directions between the throwing and the pointing group during pre-tests. We also compared movement direction alterations between groups as the difference between pre-tests and post-tests pointing movement directions. Finally, we also compared movement direction alterations between early tests and post-tests in the pointing group. Additionally, we analyzed the temporal dynamics of after-effects in early tests and post-tests for both groups. T-tests were performed to compare the mean deviations in the five first trials and in the five last trials between the throwing and the pointing group.

## Supplementary information

Supplementary figures.
