# Supplementary material for: Inter-task transfer of prism adaptation depends on exposed task mastery
Source: Sci Rep. 2020 Mar 30;10:5687. doi: 10.1038/s41598-020-62519-5 (PMC7105469; doi:10.1038/s41598-020-62519-5)
Supplement: Supplementary file 1 — Supplementary figures. [file 41598_2020_62519_MOESM1_ESM.pdf]

Inter-task transfer of prism adaptation depends on exposed task mastery

Lisa Fleury[1,2,3], Damien Pastor[1], Patrice Revol[1,2], Ludovic Delporte[1,2], Yves Rossetti[1,2,3]

[1]INSERM U1028 CNRS UMR 5292, Imp*Act* Team, Lyon Neuroscience Research Center (CRNL), 69500 Bron, France

[2] "Mouvement et Handicap" platform, Neurological Hospital, Hospices Civils de Lyon, 69500 Bron, France

[3]Claude Bernard University of Lyon 1, 69100 Villeurbanne, France

Correspondence to: lisa.fleury@univ-lyon1.fr

Keywords: sensorimotor adaptation, prism exposure, learning, generalization, expertise, plasticity, motor behaviour

Additional information

Declaration of interest

The authors declare no competing interests.

Supplemental figures

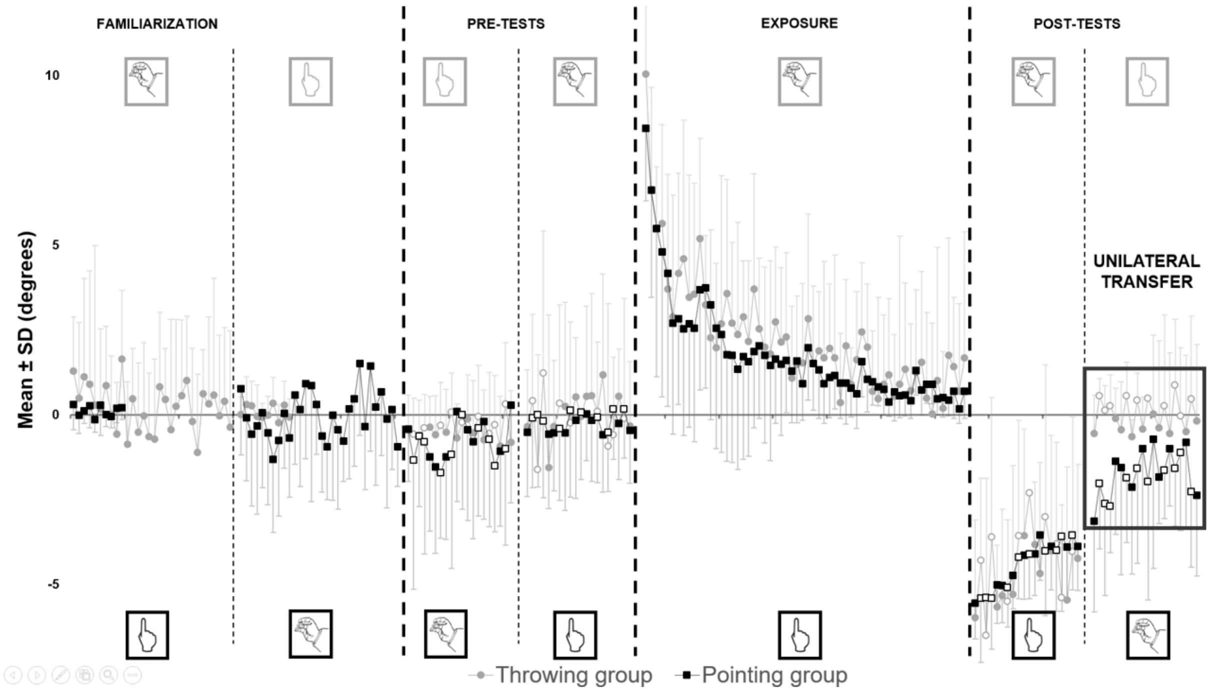

SUPPLEMENTARY FIGURE 1 – Experiment 1: trial-by-trial mean deviations. *Average group endpoint errors are illustrated for each trial during the whole experiment as well as the standard deviation (errors bars). Light grey curve and round marks refer to the throwing group, dark grey curve and square marks to the pointing group. Filled marks represent trials toward the central target and empty marks represent trials to the right target. Post-tests trials are represented with baselines subtracted.*

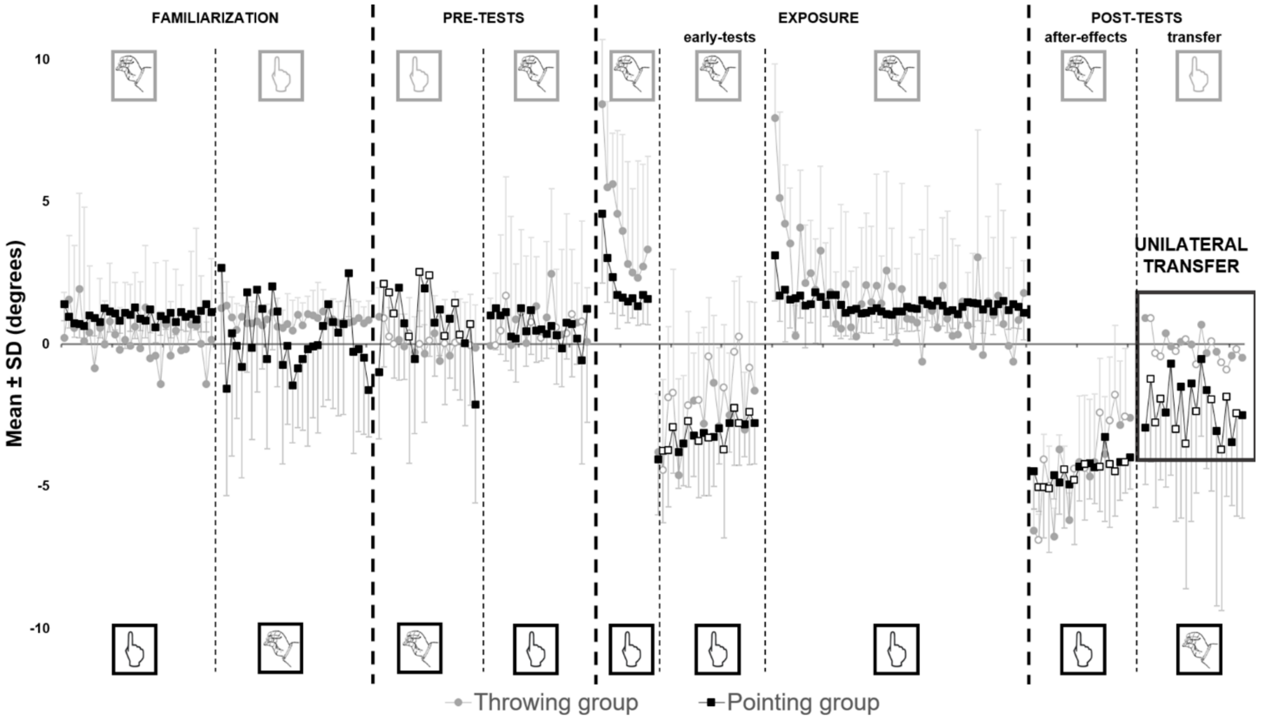

SUPPLEMENTARY FIGURE 2 – Experiment 3: trial-by-trial mean deviations. *Average group endpoint errors are illustrated for each trial during the whole experiment as well as the standard deviation (errors bars). Light grey curve and round marks refer to the throwing group, dark grey curve and square marks to the pointing group. Filled marks represent trials toward the central target and empty marks represent trials to the right target. Post-tests trials are represented with baselines subtracted.*

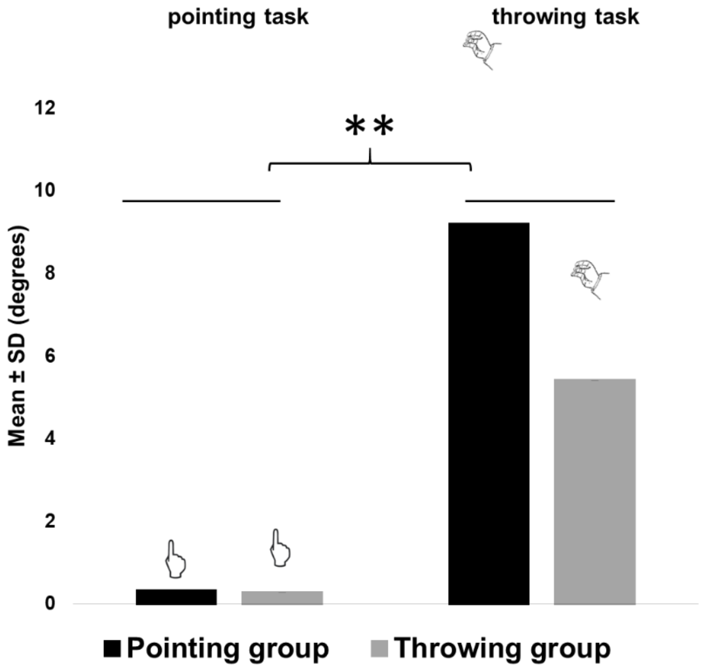

Supplementary Figure 3 – Experiment 3: mean variances during familiarization. *Mean group variances are represented in black for the pointing group, in grey for the throwing group, and respectively on the left for the pointing task and on the right for the throwing task. ** p<.01*

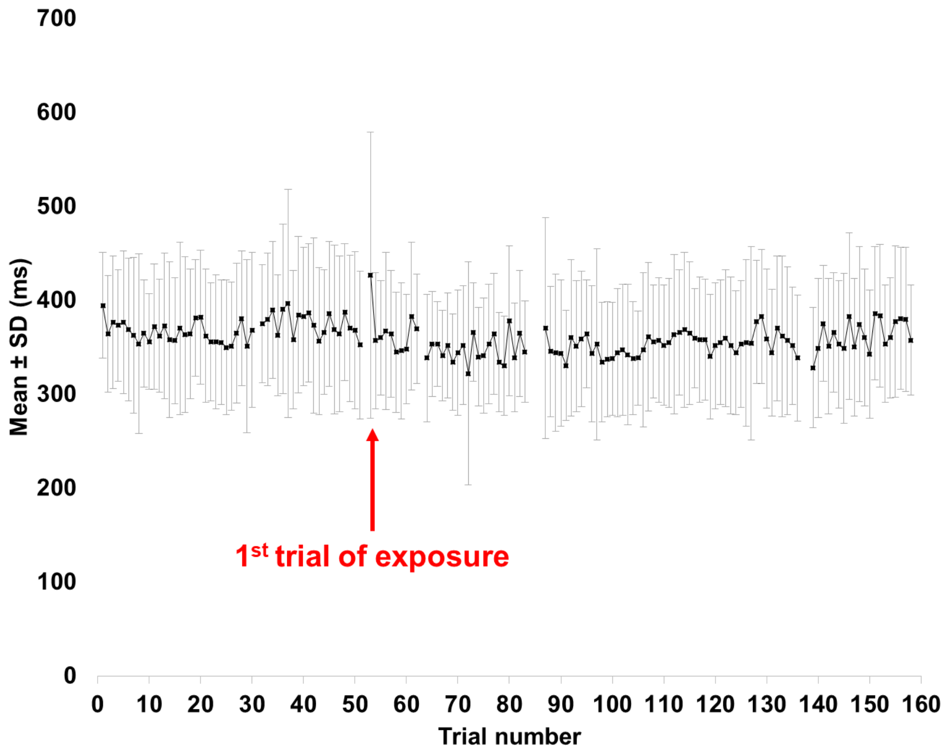

SUPPLEMENTARY FIGURE 4 – Experiment 3: trial-by-trial mean pointing movement durations in the pointing group. *Average group movement durations are illustrated for each trial during the whole experiment as well as the standard deviation (errors bars).*

A

Pointing group

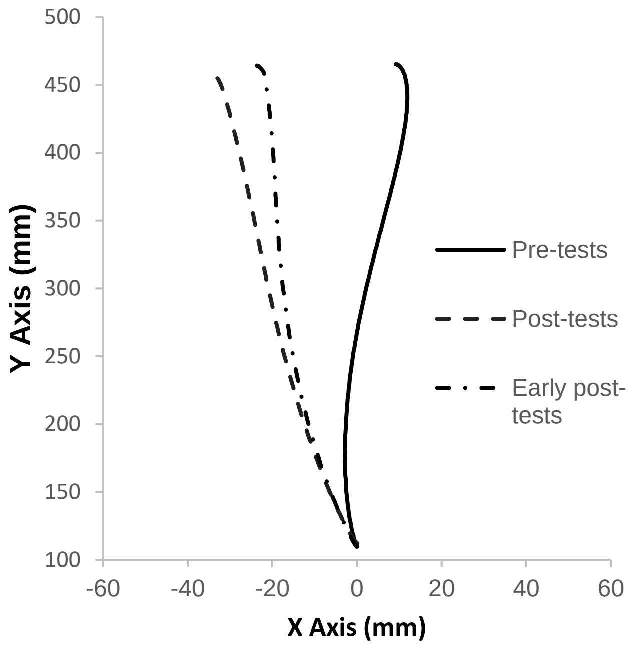

B

Throwing group

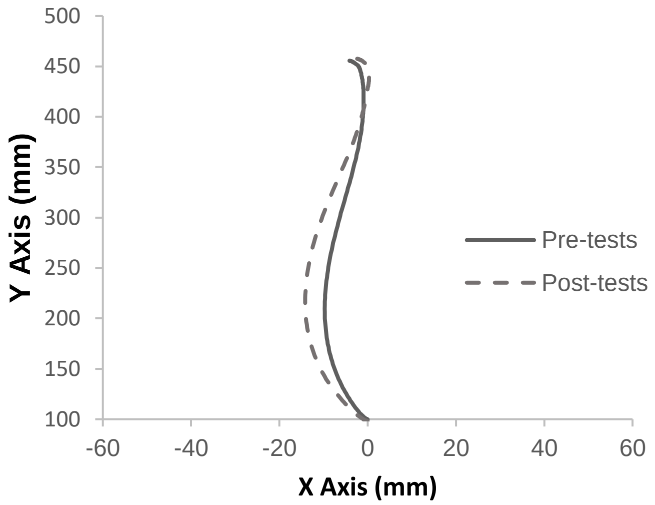

Supplementary Figure 5 – Experiment 3: mean pointing trajectories. *Mean reaching trajectories are illustrated in black for the pointing group (A; Early tests, Pre-tests and Post-tests) and in grey for the throwing group (B; Pre-tests and Post-tests only).*

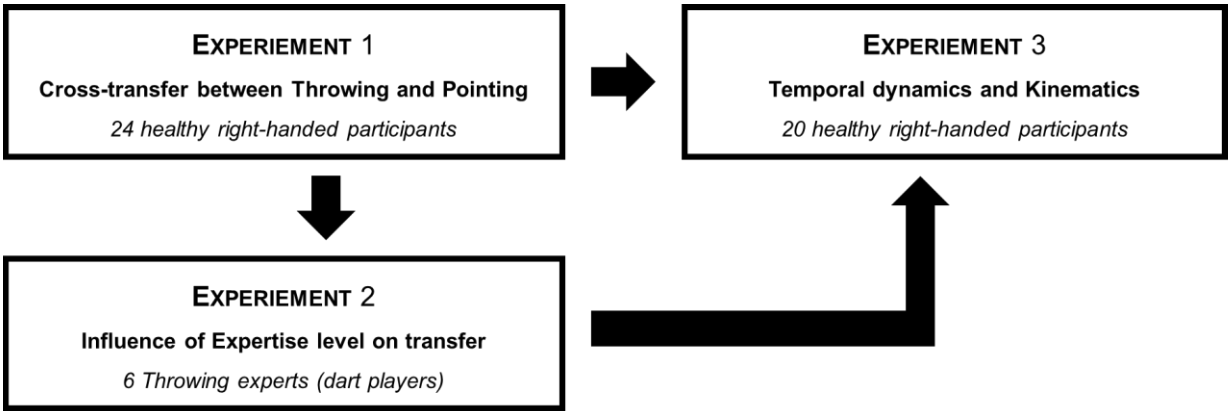

SUPPLEMENTARY FIGURE 6: Summary of the study design

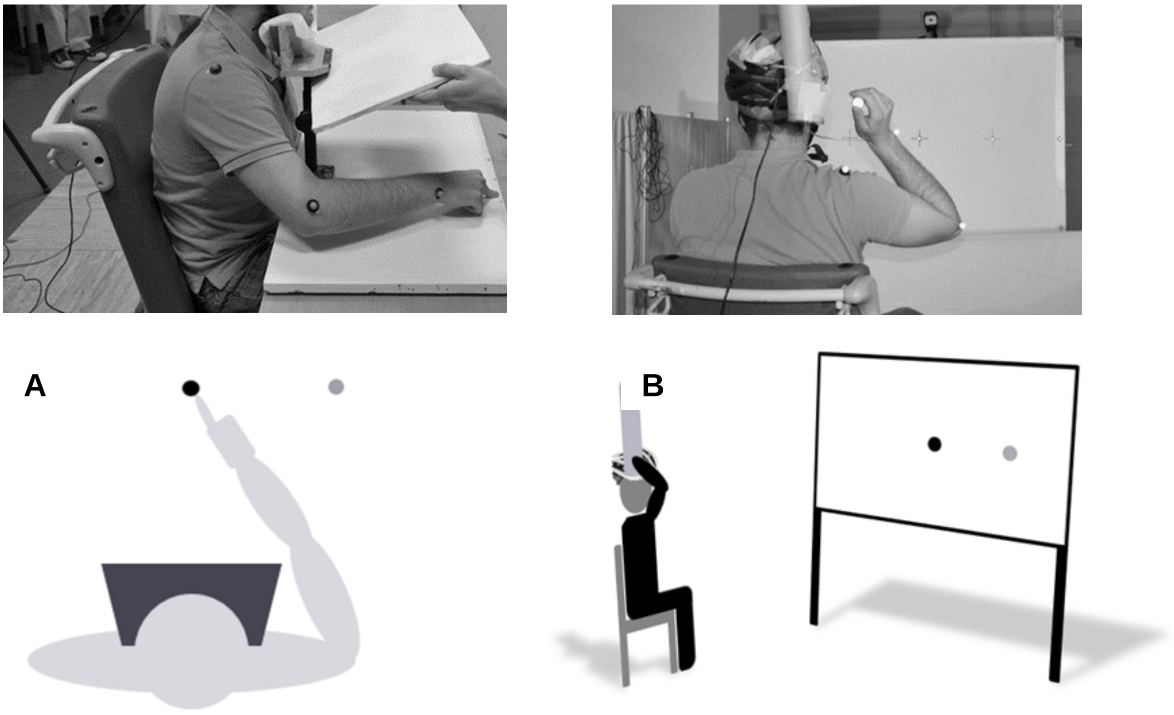

SUPPLEMENTARY FIGURE 7: Illustrations of the experimental set-up for pointing (A) and throwing (B) tasks. *Picture of the pointing task refers to open-loop condition in experiment 1 and 2.*
